# Characterization of CYP125A13, the First Steroid C-27 Monooxygenase from *Streptomyces peucetius* ATCC27952

**DOI:** 10.4014/jmb.2007.07004

**Published:** 2020-09-15

**Authors:** Hemraj Rimal, Pradeep Subedi, Ki -Hwa Kim, Hyun Park, Jun Hyuck Lee, Tae-Jin Oh

**Affiliations:** 1 Department of Life Science and Biochemical Engineering, Graduate School, SunMoon University, Asan 3460, Republic of Korea; 2 Division of Biotechnolog_y_, College of Life Sciences and Biotechnolog_y_, Korea University, Seoul 0841, Republic of Korea; 3 Unit of Research for Practical Application, Korea Polar Research Institute, Incheon 21990, Republic of Korea; 4 Department of Polar Sciences, University of Science and Technolog_y_, Incheon 21990, Republic of Korea; 5 Genome-based BioIT Convergence Institute, Asan 31460, Republic of Korea; 6 Department of Pharmaceutical Engineering and Biotechnolog_y_, SunMoon University, Asan 3140, Republic of Korea

**Keywords:** *Streptomyces peucetius*, cytochrome P450, CYP125A13, 27-hydroxycholesterol, regio-selective hydroxylation

## Abstract

The characterization of cytochrome P450 CYP125A13 from *Streptomyces peucetius* was conducted using cholesterol as the sole substrate. The in vitro enzymatic assay utilizing putidaredoxin and putidaredoxin reductase from *Pseudomonas putida* revealed that CYP125A13 bound cholesterol and hydroxylated it. The calculated K_D_ value, catalytic conversion rates, and *K*_m_ value were 56.92 ± 11.28 μM, 1.95 nmol min^−1^ nmol^−1^, and 11.3 ± 2.8 μM, respectively. Gas chromatography-mass spectrometry (GC-MS) analysis showed that carbon 27 of the cholesterol side-chain was hydroxylated, characterizing CYP125A13 as steroid C27-hydroxylase. The homology modeling and docking results also revealed the binding of cholesterol to the active site, facilitated by the hydrophobic amino acids and position of the C27-methyl group near heme. This orientation was favorable for the hydroxylation of the C27-methyl group, supporting the in vitro analysis. This was the first reported case of the hydroxylation of cholesterol at the C-27 position by *Streptomyces* P450. This study also established the catalytic function of CYP125A13 and provides a solid basis for further studies related to the catabolic potential of *Streptomyces* species.

## Introduction

During catalytic reactions, cytochrome P450 monooxygenase (P450) utilizes molecular oxygen to insert an oxygen atom into the substrate, which is usually an organic molecule. Simultaneously, the remaining oxygen atom is reduced to water [1]. The main feature of P450 enzymes is the hydroxylation of the carbon atoms of hydrophobic substrates. Similarly, the variety and flexibility of P450 enzymes make them nature’s precious gift to a variety of chemical reactions [2]. They catalyze various types of chemical reactions like C-hydroxylation, heteroatom oxygenation, dealkylations, and epoxidations, making them appealing and versatile enzymes to produce various compounds with cosmetic and pharmaceutical value [3-5]. Most bacterial cytochrome P450s (CYPs) depend on electron transfer components (redox partners) comprised of NAD(P)H-dependent ferredoxin reductase (FDR) and ferredoxin (FDX), which sequentially deliver electrons from the cofactor to heme iron during catalysis [6]. Moreover, some CYPs such as peroxygenases from the CYP152 P450 family function efficiently, using hydrogen peroxide (H_2_O_2_) without the need for redox partners [7, 8].

**Fig. 1:**
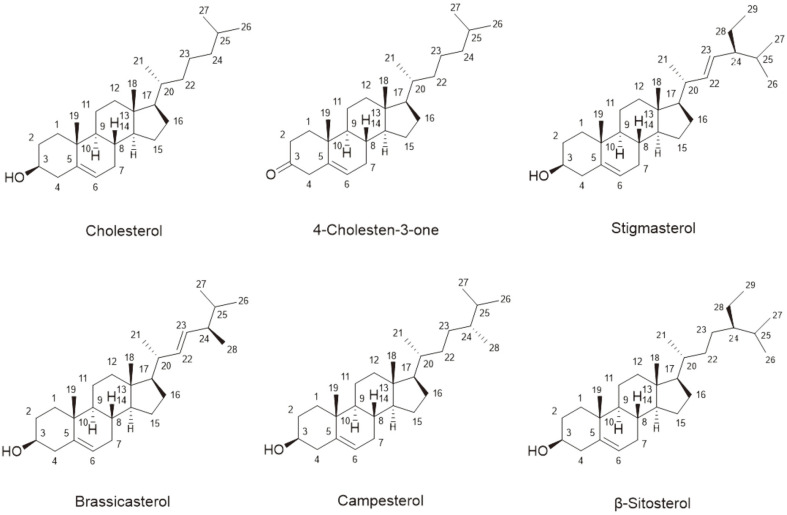
Various structures of sterols found in nature, with the carbon atom positions numbered. Cholesterol is a common sterol in animals. 4-Cholesten-3-one has a ketone at the C3-position. Sitosterol, campesterol, and stigmasterol are common phytosterols. Stigmasterol contains a double bond at the C22-position. Brassicasterol is a 28-carbon sterol synthesized by several unicellular algae including some plants.

Examination of the genome sequence showed that *Actinobacteria* contained a large number of genes representing P450s, relative to other prokaryotes [9]. There are 20 P450 genes in *Streptomyces peucetius* alone. The exact function of most of these monooxygenases is still unknown. However, some are assumed to be involved in steroids and sterol (cholesterol) metabolism (Fig. 1). Cholesterol is an important compound, essential for cell membrane structure and fluidity, steroid hormone synthesis, and bile production in humans [10]. In the case of microorganisms, *Mycobacterium tuberculosis* (Mtb) contains many genes responsible for cholesterol metabolism. Mtb degrades cholesterol and utilizes it either for energy or as a biosynthetic precursor [11]. Mycobacterial Cyp125 (Rv3545c) is responsible for the hydroxylation of C27-steroids [12], and similarly CYP125 from Rhodococcus jostii RHA1 is a cholesterol hydroxylase orthologue to Mtb P450 (RV3545c) [13]. Cholesterol degradation in microbes involves two processes, steroid ring-opening and side-chain elimination [14]. Initially, it was assumed that ring oxidation was the first step in cholesterol catabolism in prokaryotes [15-17]. Whether ring oxidation or sterol side-chain oxidation occurs first depends upon the type of bacteria [13]. Sterol ring opening is initiated by the formation of 4-cholestene-3-one with the help of cholesterol oxidase or 3β-hydroxysteroid dehydrogenase through oxidation of the cholesterol 3β-hydroxyl group and the subsequent isomerization of Δ5 into Δ4 [15-17]. Further, the oxidation of rings A and B occurs by ring-degrading enzymes 3-ketosteroid 9α-hydroxylase and 3-ketosteroid dehydrogenase [18, 19]. Side-chain elimination occurs via hydroxylation at the C26 position followed by further oxidation of the terminal acid. Additional degradation of this terminal acid occurs by a process similar to β-oxidation [20-24].

This study reports the expression, isolation, purification, and characterization of CYP125A13 as steroid 27-monoxygnease. CYP125A13 was heterologously expressed using *E. coli* and purified. A substrate-binding assay and in vitro reactions with cholesterol and choleston-4-ene-3-one were performed. These results offer clues regarding the sterol catabolism by *S. peucetius* as the degradation of cholesterol was initiated by side-chain oxidation. This is the first report in *Streptomyces* bacterial species.

## Materials and Methods

### Chemical and Enzymes

All the steroids, sterol compounds, and the derivatization reagent N, O-bis (trimethylsilyl) trifluoroacetamide (BSTFA) were purchased from Sigma-Aldrich (USA) or Tokyo Chemical Industry (Japan). Restriction enzymes, ligase, and polymerase were purchased from Takara (Japan). All of the products purchased were of the highest grade and were obtained from commercial sources.

### Phylogenetic Tree and Multiple Sequence Alignment

Phylogenetic tree analysis of CYP125A13 (NCBI gene accession number, AJ605549) was achieved using Molecular Evolutionary Genetics Analysis version 7.0 (MEGA7) [25]. The distance was kept as a two-parameter model, and clustering was done by the neighbor-joining method and analyzed using bootstrap values based on 1000 replications [26]. Multiple sequence alignment was performed using GeneDoc [27] and ClustalW [28]. Similarly, for nucleotide and amino acid comparison and analysis, the National Center for Biotechnology Information (NCBI) Basic Local Alignment Search Tools (BLAST) were used.

### Bacterial Strains, Plasmids, and Growth Conditions

*E. coli* XL1-Blue MRF’ (Stratagene, USA) was used for the cloning hosts, and *E. coli* BL21(DE3) was used for the expression hosts. Luria-Bertani (LB) agar or broth was used for the growth of *E. coli*. Approved protocols were followed for all DNA manipulations such as ligations and enzymatic digestions.

### Cloning and Over-Expression of CYP125A13

Forward and reverse oligonucleotide primers (Geno-Tech, Korea) 5’- GAA TTC ATG TCC TGC CCC CAT CTG -3’ (EcoRI) and 5’- AAG CTT TCA CCC GG TCG TCA CCT GGA GT -3’) (HindIII), respectively were designed. Polymerase chain reaction (PCR) was carried out with an initial denaturation at 94°C for 7 min, 30 cycles of 94°C for 1 min, annealing at 67°C for 1 min, polymerization at 72°C for 1 min, and a final extension at 72°C for 7 min. The pGEM-T Easy vector was used for cloning the purified PCR product, with successive transformation into *E. coli* XL1-Blue for DNA amplification. The genes were sequenced to identify errors that may have occurred during the PCR. The gene was then digested with the respective endonuclease enzymes (EcoRI and HindIII) and separated from the T-Vector for insertion into the previously liberalized pET28a(+) and pET32a(+) expression vectors. The final DNA constructs bearing an N-terminal 6xHis-tag and an isopropyl-β-D-thiogalactoside (IPTG)-inducible T7 phage promoter were transformed into the competent *E. coli* BL21 (DE3) and C41 expression hosts. The transformed colonies were selected on LB agar plates containing antibiotics. For protein expression, 50 ml of LB-medium with antibiotics was inoculated with 1.0 ml of an overnight-grown seed culture from an individual colony and incubated at 37°C in an orbital shaker (180 rpm). When the cell density reached 0.6 at OD_600_, 0.5 mM of FeCl_3_ and 1 mM of α-aminolevulinic acid (ALA) were added to the culture. After incubation for 20 min at 20 °C, the cells were induced by the addition of 1 mM IPTG and incubated for 48 h at 20°C. The cell pellets were collected by centrifugation at 3,000 ×*g* for 15 min and washed twice with 50 mM Tris-HCl buffer (pH 7.4) containing 15% glycerol. The cell pellets were mixed in 1.0 ml of the same buffer and homogenized by a French press. The soluble protein fraction was separated from the insoluble cell fraction by centrifugation at 12,000 rpm for 30 min at 4°C. Finally, the protein obtained was examined by 12% sodium dodecyl sulfate-polyacrylamide gel electrophoresis (SDS-PAGE).

### Expression of Redox Partners

To examine the electron transport system of CYP125A13, the over-expression of putidaredoxin (PDX) and putidaredoxin reductase (PDR) was carried as follows. Pdx and PdR were ligated into pET28a(+) and pET32a(+) vectors as follows. The pETDuet-P450cam/Pdx plasmid was used to excise Pdx by restriction enzymes NdeI and XhoI and ligated to previously linearized pET28a(+) as described [29]. Similarly, PdR was excised from the pACYDuet-PdR vector using restriction enzymes NcoI and XhoI and ligated to the pET32a(+) vector. The resulting DNA constructs pET28a-Pdx and pET32a(+)-PdR encoded N-terminally 6xHis-Tagged Pdx and PdR [3]. The DNA constructs were transformed into a competent cloning and expression host with a subsequent screening on LB-agar supplemented with antibiotics. For recombinant Pdx and PdR expression by *E. coli* BL21 (DE3) cells harboring the DNA construct, a procedure similar to the expression of CYP125A13 mentioned above was performed.

### CO-Reduction Assay

The CO-reduction assay of CYP125A13 was conducted according to a defined protocol [30]. The protein was diluted in potassium phosphate buffer (50 mM) containing 10% glycerol. A small amount of sodium dithionite was added and the solution was divided into two cuvettes. One cuvette was used as a reference while the other was saturated with 60–70 CO bubbles at a rate of one bubble per second. The solution was then scanned between 400 and 500 nm at room temperature using a Shimadzu 1601PC spectrophotometer. The CYP concentration was calculated by the difference in the absorbance at 450 nm and 490 nm using an extinction coefficient of 91 mm^-1^cm^-1^.

### Electron Paramagnetic Resonance (EPR) Measurement

To record the CYP125A13 electron paramagnetic resonance (EPR) spectra, a Bruker X-band spectrometer (9.5 GHz) equipped with a continuous supply of helium cryostat ESR 900 and an ITC 4 temperature controller used to achieve a base temperature of 5 K, was used. The microwave frequency and modulation frequency used were 9.647GHz and 100 kHz, respectively. The data were recorded and analyzed at The Korea Basic Science Research Institute, Western Seoul Center. Before the measurement, the oxidized form of 180 μM CYP12A13 in 10 mM potassium phosphate buffer (pH 7.4) was frozen in liquid nitrogen in EPR quartz tubes (Wilmad). The spectral data comprising the g-tensor and line width of the heme centers were acquired by the accumulation of spectra in the Simfonia or Xsophe (Bruker) programs.

### Substrate-Binding Assay

Substrate-binding assays were performed in 50 mM potassium phosphate buffer at pH 7.4 containing 0.1 mM EDTA with 1 μM enzyme and increasing substrate concentrations until saturation. Stock solutions of 5 mM steroids were prepared in buffer with 10% (w/v) β-methyl cyclodextrin (BMCD), and the absorbance spectra of all samples were measured using a Biochrome Libra S35PC UV/Visible Spectrophotometer (England). Difference spectra were recorded from 350 to 500 nm. The titration data points were fitted to the quadratic equation using GraphPad Prism 7.0 (GraphPad software, USA) to determine the K_D_ values [31]: A_obs_ = A_max_ (([S]+[E_t_]+K_D_) − (([S]+[E_t_]+K_D_)^2^ − (4[S][E_t_])^0.5^)/2[E_t_]. In the equation, A_obs_ was the absorption shift observed at any concentration of ligand; A_max_ was the maximal absorption shift observed at saturation; K_D_ was the apparent dissociation constant; [E_t_] was the concentration of enzyme used; and [S] was the substrate concentration.

### Kinetic Analysis

Kinetic studies were performed in a reaction system consisting of 1 μm CYP125A13, 10 μM PDX, 1 μM PDR, 100 ng catalase, and 500 μM NADH in 50 mM sodium phosphate buffer using substrate concentrations ranging from 5 to 350 μM. The product formation rate versus the substrate concentration was plotted and the *K*_m_ and *kcat* kinetic parameters of the enzyme were calculated using the Michaelis–Menten equation with GraphPad Prism by employing nonlinear regression.

### In Vitro Assay of Cholesterol to 27-Hydroxycholesterol by CYP125A13

The activity of CYP125A13 was determined using a C-27 steroid as a substrate. The reaction mixture consisted of 1 μm CYP125A13, 1 μM PDR, 10 μM PDX, 10 U formate dehydrogenase, 150 mM sodium formate, 100 ng catalase, 10 mM MgCl_2_, and 150 μM substrate in 50 mM sodium phosphate buffer. The reaction was initiated by adding 250 μM NADH. After incubating at 30°C for 2.5 h, the reaction was quenched with 100 mM Tris buffer and extracted with a double volume of diethyl ether. The diethyl ether extract was dried under nitrogen gas, derivatized with BSTFA (1% TMCS) and pyridine, and analyzed by gas chromatography-mass spectroscopy (GC-MS) [32, 33].

### Homology Modeling and Docking Study

The crystal structure of CYP125 from *Mycobacterium tuberculosis* with the cholest-4-en-3-one substrate (PDB ID: 2X5W, 1.58 Å and 55.96% sequence identity with CYP125A13) was chosen as the template [34]. A three-dimensional (3D) model of CYP125A13 was generated using Modeller 9.9.2 [35]. Validation of the homology models was done by Ramachandran plot and ProSA 2003 z-scores [36, 37]. Molecular docking was accomplished using AutoDock Vina-1.1.2 [38]. The graphical user interface program AutoDock Tools 1.5.6 was used to prepare the PDBQT files of the protein and ligand and for grid box creation [39]. A grid box of 24.5 Å × 24.5 Å × 24.5 Å with 1 Å spacing was used to prepare the grid map, covering the entire ligand molecule. All the other parameters were set to the default values. Visualization of the docked protein and ligand was performed using PyMOL [40].

## Results and Discussion

### Phylogenetic Tree and Sequence Alignment of CYP125A13

**Fig. 2:**
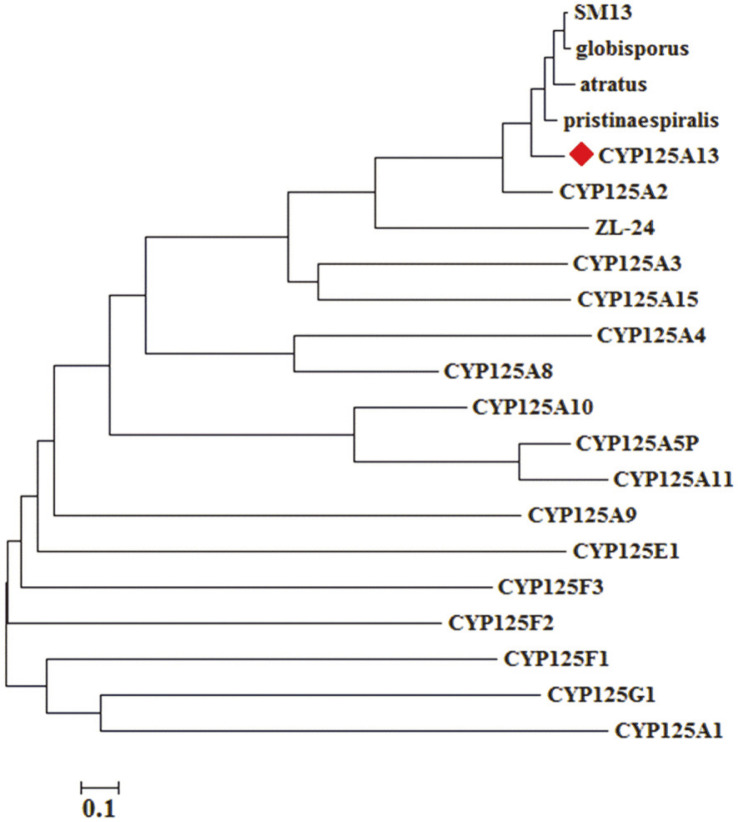
Phylogenetic tree of the heme domain of selected CYP125A family enzymes including CYP125A13 from *S. peucetius*. Multiple sequence alignment was performed using ClustalW. The phylogenetic tree was constructed by the neighbor-joining method. The bar in the lower-left corner represents 0.1 amino acid substitutions per amino acid for the branch length.

To find the similarity of CYP125 subfamily monooxygenases among various bacterial strains, the phylogenetic relationship of the heme domains was examined. The phylogenetic tree showed that CYP125A13 is clustered with other *Streptomyces* species (Fig. 2). Although the sequence correlation within the CYP125 subfamily is distinct, *S. peucetius* contains a gene which encodes a protein similar to CYP125A1 (55.96% sequence identity). BLAST was applied to find the protein identical to that encoded by CYP125A13. A comparison of the heme domains with other members of the CYP125A family showed sequence identity and similarities. The heme domain sequence alignment showed that the extremities were poorly conserved, while greater sequence identity was observed in areas important for enzymatic activity and substrate binding.

**Fig. 3:**
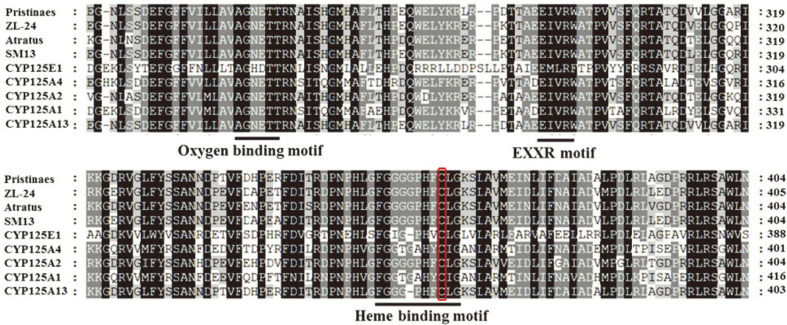
Multiple sequence alignment of CYP125A13 compared to other CYP125 family enzymes using CLUSTALW. The different conserved structural motifs including the oxygen-binding motif, EXXR motif, and the characteristic heme-binding motif of the CYP subfamily are underlined. Conserved cysteine residue of the heme-binding motif is highlighted in red. The conserved residues are indicated in bold letters.

To find the homologs of the protein sequences, a PSI-BLAST search (NCBI server) was conducted. Multiple sequence alignment was performed to identify sequence conservation and the signature motifs of CYP125A13. The conserved sequences, percent sequence identity, and chain length of CYP125A13 were compared with other CYPs. Various signature motifs in CYP125A13 were conserved. The signature motifs of CYP125A13 were the oxygen-binding motif, the EXXR (EIVR) motif, and the characteristic signature heme-binding motif for the CYP subfamily FxxGxHxCxG (FGGGPHFCLG) (Fig. 3). The signature motif showed a conserved cysteine residue ligated to the heme iron (Fe). Heme permanently bound to protein helps bind the ligands that can bind divalent iron (Fe). The bound molecules can modulate the function of the P450s. CYP125A13 in the substrate-binding pocket (Fig. 7B) also shared key amino acid residues with eukaryotic CYPs belonging to various families involved in cholesterol metabolism, suggesting sterols as a substrate for CYP125A13. A similar observation for the C-26 hydroxylation of the sterol side chain in *Rhodococcud jostii* RHA1 was reported by Rosloniec *et al*. [13].

### Cloning, Over-Expression of CYP125A13, and CO-Reduction Assay

**Fig. 4:**
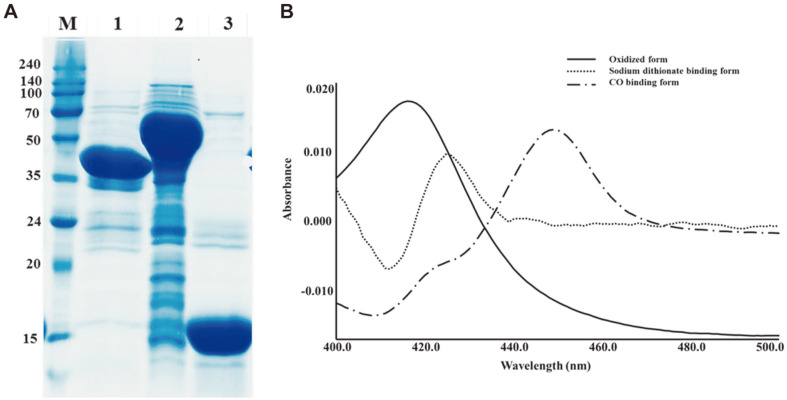
Purification of His-tagged CYP125A13 and spectroscopic characterization. (**A**) SDS-PAGE analysis of purified CYP125A13 (lane 1, 46.6 kDa), PDR (lane 2, 58 kDa), and PDX (lane 3, 10 kDa) where M is the standard protein marker. (**B**) Typical CO-difference spectra of CYP125A13. The solid line represents the oxidized form. The dotted line represents the sodium dithionite-bound form and the dot-dash line denotes the CO-reducd form.

*E. coli* BL21 (DE3) cells showed better yields of heterologously over-expressed CYP125A13. CYP125A13 (lane1, 46.6 kDa), PDR (lane 2, 58 kDa), and PDX (lane 3, 10 kDa) were separated and analyzed by SDS-PAGE (Fig. 4A). A cytosolic fraction obtained from heterologously expressed CYP125A13 displayed spectral properties characteristic of P450 enzymes by UV-Vis spectroscopy. The major Soret band of CYP125A13 showed a peak maximum at 420 nm, indicating the presence of a protein-heme complex in the LS state. The spectra of the carbon monoxide-bound forms exhibited a typical peak with a maximum at 450 nm (Fig. 4B), which is indicative of the native FeII-CO complexes of P450s [41].

### EPR Spectroscopy of CYP125A13

**Fig. 5:**
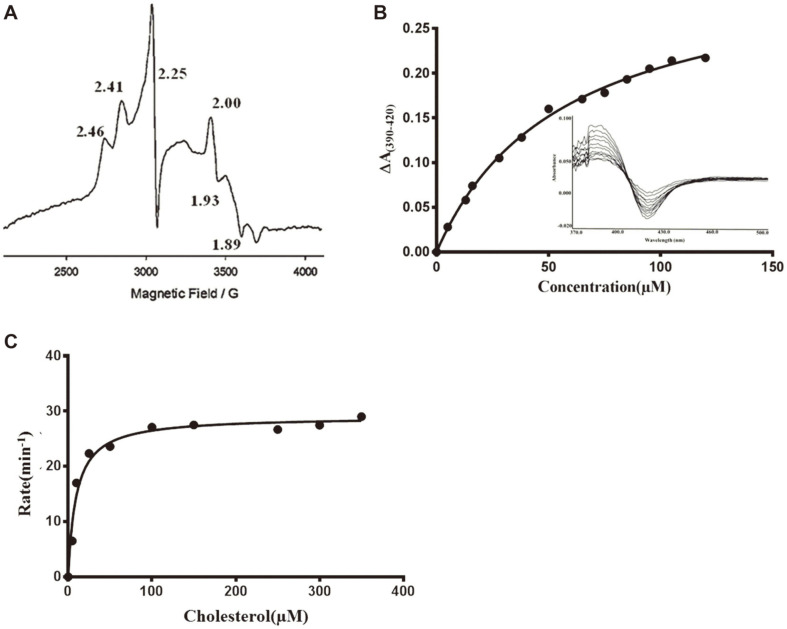
Spectroscopic characterization of CYP125A13. (**A**) The X-band electron paramagnetic resonance (EPR) spectra of oxidized CYP125A13. The apparent g values are indicated in the spectrum. (**B**) Spectral shift induced by the binding of cholesterol. Varying concentrations of substrate titrated against P450. The plot of absorbance change vs. substrate concentration from the titration is shown. Each point in the titration was plotted to a tight binding quadratic equation as described in the Experimental Procedures. (**C**) The Michaelis-Menten curve of CYP125A13 was obtained by plotting the rate of the reaction vs. cholesterol concentrations ranging from 5–350 μM.

The EPR spectra for native CYP125A13 were collected in the oxidized form to further characterize the coordinated heme in CYP125A13. Fig. 5A shows the X-band of the EPR spectra in the oxidized form, where a characteristic P450 rhombic spectrum was observed. Iron (Fe) is identified in EPR spectroscopy by the presence of unpaired or odd numbers of electrons in the resting or intermediate reactive species of metalloproteins such as cytochrome P450s because every metal has a specific nuclear spin. The EPR signal can be detected either in the reduced or oxidized form or in both forms and provides a clue to the type of metal ion present in the enzyme. Similarly, the g-values indicate whether or not a protein is in a high-spin state [42]. CYP125A13 without a ligand has g-values of g_z_ = 2.416/2.41, g_y_ = 2.25, and g_x_ = 1.93, consistent with an LS thiolate-coordinated P450 protein, and comparable to spectra already reported for the heme domain of the well-studied class III CYP102A1 from *B. megaterium* (2.42, 2.26, and 1.92 for g_z_, g_y_, and g_x_, respectively) and the class I cytochrome P450 *Mycobacterium tuberculosis* cholesterol 27-hydroxylase CYP125 (2.40, 2.25, and 1.94 for g_z_, g_y_, and g_x_, respectively) [15, 16, 43, 44]. The presence of the three major peaks indicates a nuclear spin state of ½ (2I + 1). The final g_z_, g_y_, and gx values indicated typical low-spin ferric (Fe III) heme (Fig. 5A).

### CYP125A13 Substrate-Binding Assay, In Vitro Activity Assay, and Steady-State Sterol Kinetics

CYP125A13 displayed spectral properties characteristic of P450 enzymes in UV-Vis absorption spectroscopy. CYP125A13 exhibited the spectral properties of a ferric (III) form with the majority of the heme iron occupying a high spin state with a Soret band at 390 nm. Substrate binding to the active P450 site typically results in a type I-shift of the Soret band when the water molecule is displaced relative to the heme iron [45, 46]. Both 4-choletsten-3-one and cholesterol prepared in ethanol induced significant type-I spectral changes with the introduction of CYP. To increase the solubility of hydrophobic sterol, 15% (w/v) β-methyl cyclodextrin (MBCD) was used. Stock solutions (5 mM) of both cholesterol and 4-cholesten-3-one were prepared in 10% (w/v) MBCD. The addition of cholesterol and 4-cholesten-3-one to CYP125A13 showed an almost complete conversion to the high-spin form. The K_D_ values for the C27-steroid were obtained from the spectral titration curves (Fig. 5B). The titration plots were fitted to a tight binding quadratic equation to obtain the K_D_ values of 59.23 ± 3.9 and 56.92 ± 11.28 μM for 4-cholesten-3-one and cholesterol, respectively. Capyk *et al*. [12] reported K_D_ values for 4-cholesten-3-one and cholesterol of 0.27 ± 0.05 μM and 0.20 ± 0.02 μM, respectively, for the mycobacterial Cyp125 catalysis of the terminal hydroxylation of C27-steroids. Similarly, Rosłoniec *et al*. [13] reported K_D_ values of 0.20 ± 0.03 μM for 4-cholesten-3-one and 0.20 ± 0.08 μM for cholesterol for C26-hydroxylation in *Rhodococcus jostii*. These K_D_ values are very low compared to the calculated value, suggesting a weaker binding affinity of the enzyme for the substrate. Fig. 5C shows the hyperbolic dependence of the NADH oxidation rate on cholesterol concentration in a reaction mixture consisting of 1:10:3 ratios of P450:PDX:PDR, with an apparent *K*_m_ of 11.3 ± 2.8 μM for cholesterol and a Vmax of 27.9 ± 1.5 min^-1^, considering the background rate of NADH oxidation in the absence of substrate.

**Fig. 6:**
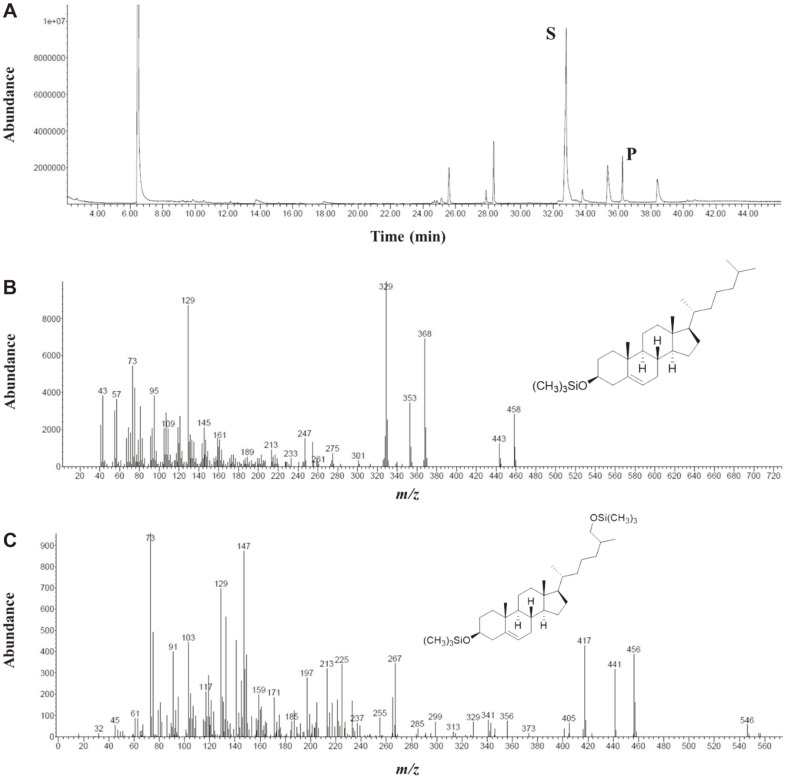
Gas chromatography-mass spectrometry (GC-MS) analysis of the hydroxylation of cholesterol by CYP125A13. (**A**) GC chromatogram of the hydroxylation of cholesterol, where S represents the substrate and P represents the 27-hydroxylated product. (**B**) Mass spectrum of cholesterol. TMS-ether of cholesterol [M]^+^ = *m/z* 458. (**C**) Mass spectrum of the product peak (27-hydroxycholesterol) from the in vitro reaction. TMS-ether of 27-hydroxycholesterol [M]^+^ = *m/z* 546.

The in vitro enzymatic assay was performed using the heterologous electron donor partners PDX and PDR as auxiliary proteins and an NADH-regenerating system. After 2.5 h of incubation with CYP125A13, cholesterol oxidation displayed a new peak in the GC chromatogram (Fig. 6A). The *m/z* value of 546 (-CH_2_OH) suggested that cholesterol was converted to its hydroxylated product (Figs. 6B and 6C). The presence of *m/z* 456, *m/z* 417, and *m/z* 73 in the mass spectra confirmed the identification of the compound as 27-hydoxycholesterol. Similar *m/z* values were reported by McLean *et al*. [44] for 27-hydroxycholesterol. Confirmation of the product was made after the mass values exactly matched the trimethylsilyl (TMS)-derivatized 27-hydroxycholesterol standard in the GC mass data bank in *The Wiley/NBS Registry of Mass Spectral Data 7th edition* [47]. The catalytic conversion rate of cholesterol at a substrate concentration of 50 μM was determined to be 1.95 nmol min^−1^ nmol^−1^ CYP. Surprisingly, no product formation was detected with 4-cholesten-3-one. The human P450 isoform CYP7A1 showed increased enzymatic activity toward cholesterol after the addition of ionic Tween-20 detergent [48]. When Tween-20 was substituted with MBCD no activity was observed for either substrate. Despite some observation of NADH oxidation, the lack of cholesterol solubility in aqueous buffer hindered the accurate determination of the catalytic parameters.

### Homology Modeling and Docking Study

The 3D protein models were generated by Modeller using the most homologous template 2X5W from *Mycobacterium tuberculosis*. To validate the quality of the homology model, the Ramachandran plot and ProSA were utilized. In the Ramachandran plot, distribution of the backbone dihedral angles ψ and φ in the amino acid residues of the refined structure revealed that 95.6% were in the favored region, 3.2% were in the allowed region, and 1.2% were in the outlier region (Fig. S1A). In addition, the z-score value of -10.38 showed the structure to be within the score range of native proteins of similar sizes from different sources (X-ray and NMR) (Fig. S1B), indicating the acceptability and trustworthiness of the optimized model.

**Fig. 7:**
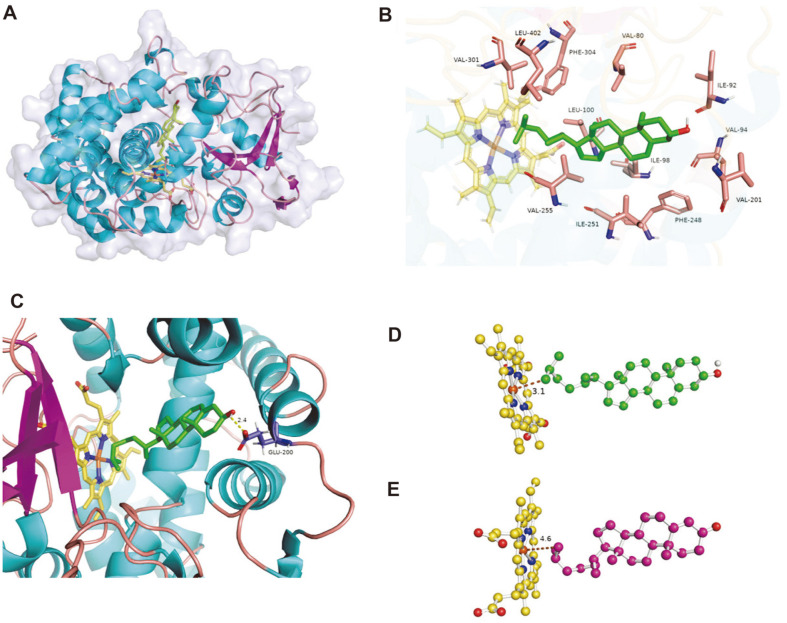
Structural analysis of the CYP125A13-substrate complex. (**A**) Ribbon representation of the homology model of CYP125A13 with bound cholesterol (green). (**B**) Amino acid residues surrounding the cholesterol in the active site of the protein. (**C**) Hydrogen bonding between the C3-OH of cholesterol and the carboxyl group of the Glu200 residue. Ball and stick representation of the aliphatic C27 of cholesterol (**D**) and 4-cholesten-3-one (**E**) facing the heme within the active site with distances of 3.1 Å and 4.6 Å, respectively. The brown line represents the distance between the substrate and the heme porphyrin. The cholesterol and 4-cholesten-3-one are represented in green and magenta, respectively.

The modeled 3D structure of CYP125A13 showed a large pocket just above the heme porphyrin that could potentially accommodate C27-sterols (Fig. 7A). The substrate-binding domain showed a variety of topologies, which explained the substrate preference. The substrate-binding pocket was funnel-like in shape and the shape narrowed down toward the catalytic site to enclose the aliphatic side chain of the ligand. Partially conserved hydrophobic residues, which are expected to interact with the buried substrate, occupied the internal cavity. Molecular docking was performed using AutoDock Vina to access the binding position of cholesterol and 4-cholesten-3-one to CYP125A13 (Figs. S2A and S2B). Two types of docked configurations were observed for both steroids, with allylic C27 facing toward and away from the heme center (Figs. S2C and S2D). The configurations with allylic C27 facing toward the heme center predominated and among them, the configuration with the lowest energy conformation was selected for further analysis. The docking result showed that cholesterol and 4-cholesten-3-one were deeply buried in the CYP125 active site, with the aliphatic side chain containing terminal methyl groups (C27) facing the distal surface of the heme (at distances of 3.1 and 4.6 A, respectively) (Figs. 7D and 7E). This orientation of cholesterol and 4-cholesten-3-one was stabilized by hydrophobic amino acids like Val80, Ile92, Ile98, Val94, Leu100, Val201, V255, Ile251, Phe248, Val301, Phe304, and Leu402 (Fig. 7B). McLean *et al*.[44] reported that in CYP125, the alkyl chain is in close contact with the Val267 residue, which is required for the low-high spin-state transition. The multiple sequence alignment and docking study showed that Val255 in CYP125A13 corresponded to Val267, interacting with the substrate and that it might play a crucial role in spin state transition. In addition, Johnston *et al*. [49] reported that the hydroxylation of a methyl group in phytanic acid was the result of the tight binding of the substrate and many weak interactions within the active site. In this study, binding of the tetracyclic portion of sterol in the active site, in addition to the binding of the aliphatic side-chain along with one methyl group in the hydrophobic cavity of the active site, locked the other methyl in position for the hydroxylation, yielding region-specific C27-hydroxylation. Hydrogen bonding (2.4 Å) was observed between the hydroxyl group of cholesterol and the carboxyl group of Glu200 in the docked model (Fig. 7C). The hydrophilic residues surrounded the keto or hydroxyl group of C3 by forming hydrogen bonds or hydrogen bonding networks through water molecules, stabilizing the ligand.

## Conclusion

The present study provided a biochemical characterization of CYP125A13, a 27-monooxygenase of cholesterol with putative roles in (i) cholesterol catabolism and (ii) the 3-step oxidative transformation to the 27-acid or (ii) the 27-hydroxylated product by a single P450 turnover. An in vitro assay was used to characterize the molecular function and physiological role of CYP125A13. A comparative homology model was built, refined, and validated. Docking of the C27-steroid on the homology model showed that all the residues engaged in the binding of the C27-steroid were located in substrate recognition sites. The binding studies along with the in vitro enzymatic activity revealed that CYP125A13 could utilize cholesterol as a substrate for hydroxylation. This study provides new insight into the initiation of cholesterol degradation in *S. peucetius*. The CYP125A13 findings suggest that *S. peucetius* may be involved in extra C27-oxidase activity that transforms 27-hydroxycholesterol to other carboxylic acid derivatives and a similar enzyme responsible for side-chain degradation. This essential step is needed for the initiation of degradation of the ring nucleus. The search for other genes responsible for ring oxidation in *S. peucetius* is underway. Further study of these important initial steps of cholesterol hydroxylation will lead to a better understanding of sterol catabolism in *S. peucetius*.

## Supplemental Materials



Supplementary data for this paper are available on-line only at http://jmb.or.kr.
