# Supplementary material for: Characterization of CYP125A13, the First Steroid C-27 Monooxygenase from Streptomyces peucetius ATCC27952
Source: J Microbiol Biotechnol. 2020 Sep 15;30(11):1750–9. doi: 10.4014/jmb.2007.07004 (PMC9728343; doi:10.4014/jmb.2007.07004)

J. Microbiol. Biotechnol.
https://doi.org/10.4014/jmb.2007.07004

Fig. S1. Homology model validation by Ramachandran plot and z-scores. (**A**) Ramachandran plot of the CYP125A13 modeled structure was quantitatively analyzed and 95.6% of the values were in the most favored region, 3.2% in the allowed region, and only 1.2% in the outlier region. (**B**) The ProSA z-score of -10.38 indicates a good correlation between the modeled structure and the native structure from different sources (X-ray and NMR). The ProSA z-score assesses the quality of the model.

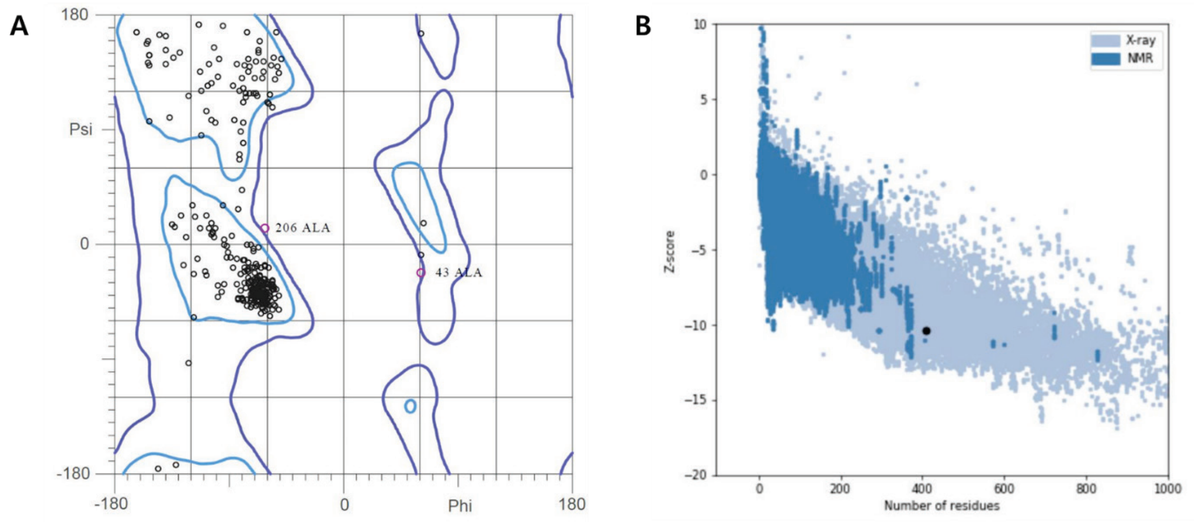

Fig. S2. Cross-sectional view of the substrate binding domain of the enzyme with bound cholesterol (**A**) and 4-cholesten-3-one (**B**) in the active site of CYP125A13. Various orientations of cholesterol (**C**) and 4-cholesten-3-one (**D**) in the active site obtained after docking. Ligand positions with allylic C27 facing toward the heme moiety were predominant.

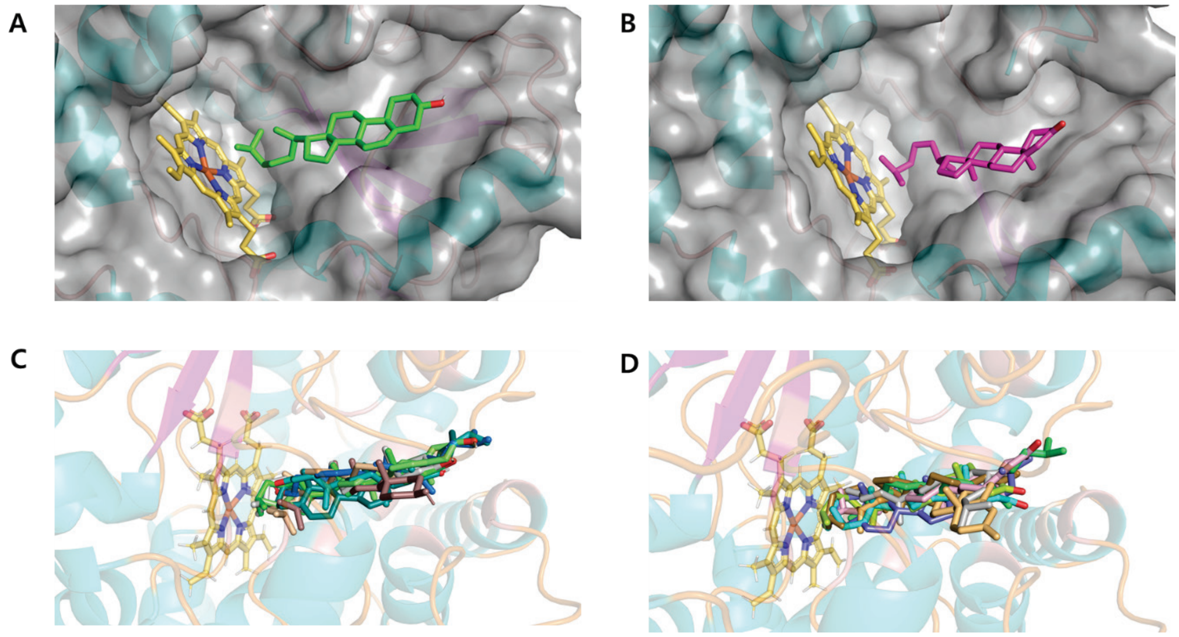

Supplement: Supplementary file 1 [file JMB-30-11-1750-supple.pdf]
